# Proteomic Identification of Novel Plasma Biomarkers and Pathobiologic Pathways in Alcoholic Acute Pancreatitis

**DOI:** 10.3389/fphys.2018.01215

**Published:** 2018-08-30

**Authors:** Richard T. Waldron, Aurelia Lugea, Aiste Gulla, Stephen J. Pandol

**Affiliations:** ^1^Pancreatic Research Group, Department of Medicine, Cedars-Sinai Medical Center, Los Angeles, CA, United States; ^2^Department of Surgery, Georgetown University Hospital, Washington, DC, United States; ^3^Department of Surgery, Vilnius University Hospital Santaros Clinics, Faculty of Medicine, Vilnius University, Vilnius, Lithuania

**Keywords:** acute pancreatitis, biomarkers, C-reactive protein, ethanol, Orbitrap Elite, proteomic, severity, transketolase

## Abstract

Acute pancreatitis (AP) is a painful and potentially life-threatening disorder with the potential for therapeutic interventions. Biomarkers that characterize cases by severity and pathogenic mechanisms involved are not yet available but needed for the implementation of rational therapies. Here, we used shotgun proteomics to obtain information from plasma samples about local and systemic pathologies taking place during cases of alcoholic AP. Plasma was obtained at Kaunas University of Medicine Hospital (Lithuania) from 12 AP patients of alcohol related etiology (median age of 40) within 24 h of presentation, and 12 age-matched, healthy controls. Patients entered into the study had moderately severe AP with the following characteristics: mean blood lactate dehydrogenase level of 1127 mg/dl; median APACHEII score of 5.5 and mean IMRIE score of 3.5. For proteomic analysis, less-abundant proteins in plasma samples were enriched using Top 12 abundant protein depletion columns. Further processing was performed by a modified filter-assisted sample preparation combined with tandem mass tag labeling for quantitation. Samples were analyzed using an Orbitrap Elite mass spectrometer for high resolution liquid chromatography–tandem mass spectrometry (LC–MS/MS). Our analysis revealed 31 proteins that exhibited significant 1.5-fold or higher increases in the AP compared to control patients, and six that were significantly decreased. Gene ontology analysis indicated a strong correlation with exosomal origin in the elevated proteins, with 29/31 (93.5%) associated with this extracellularly-secreted compartment. Elevated proteins included established and proposed biomarkers of AP including C-reactive protein, LPS-binding protein, intercellular adhesion molecule-1, and von Willebrand factor, as well as several novel potential biomarkers. These results provide the methodology for proteomic analysis of plasma samples to discover novel biomarkers that characterize pancreatitis cases by pathogenic mechanism as well as disease activity at an early stage that is highly informative for routine clinical practice and clinical trials.

## Introduction

Acute pancreatitis is one of the most common diseases of the gastrointestinal tract, leading to tremendous emotional, physical, and financial human burden (Papachristou et al., 2010). In the United States, in 2009, AP was the most common gastroenterology discharge diagnosis with a cost of 2.6 billion dollars. Recent studies show the incidence of AP varies between 4.9 and 73.4 cases per 100,000 worldwide (Tenner et al., 2013).

Alcohol consumption has been identified as a primary cause of both acute and chronic pancreatitis in most developed countries. About one-third of AP in the United States is alcohol-related, being more common in men, and the other most common cause is gallstones (Dufour and Adamson, 2003). The relative contribution of heavy alcohol consumption is likely to be greater than gallstones for RAP, although this may vary based on the demographic distribution. In Lithuania, as elsewhere in the world, the detrimental effects of alcohol consumption on health are of concern (Klumbiene et al., 2012). Alcohol does not cause pancreatitis by itself but increases the susceptibility to pancreatitis by affecting pancreatic physiology at multiple levels, of which the most important is its ability to sensitize the pancreas to other insults (Aframian et al., 1996; Pandol et al., 1999). One such cofactor is smoking, which can also increase the risk of pancreatitis independently (Andriulli et al., 2010; Greer et al., 2015). Genetic susceptibility can explain an increased risk of pancreatitis in some individuals who also drink.

Regardless of the environmental factors that accompany and modify alcohol drinkers, AP related to alcohol tends to exhibit significant organ damage and can be among the severe cases with systemic inflammatory response syndrome (SIRS) and organ failure involving the pulmonary, cardiovascular, and renal systems (Ignatavicius et al., 2017). Single and combination treatments to combat disorders in these organs have not been developed for AP. Thus, early biomarkers of impending SIRS, MODS, or infected necrosis in AP would be especially valuable.

Scoring systems such as the Ranson’s, APACHE-II, and Imrie systems have been used to help predict the severity level and course of disease, but current trends have emphasized a desire in the field to simplify these systems while retaining their predictive performance (Papachristou et al., 2010; Wu et al., 2017). Many stand-alone biomarkers have been proposed and evaluated over the years, but very few have come into routine clinical practice, either due to insufficient specificity or selectivity in the biomarker or because inexpensive tests are not readily available. CRP, as well as IL-6 have been found highly specific and selective, and an inexpensive test has come into use for CRP (Meher et al., 2015). This marker typically peaks at ∼72 h after the onset of clinical AP and then subsides in mild, but not in severe cases. Thus, CRP levels above ∼250 g/L indicate the presence of a systemic inflammatory process such as AP. Improved measures that evaluate the systemic effects of the disease and identify targets for therapeutics are needed. Some experimental therapeutic approaches are already under study and may prove beneficial, but we do not have measures to determine pathways they would affect during the disease. The ongoing development of such measures is necessary, as these will need to be applied to personalized treatment approaches.

Here, we performed a quantitative proteomic technique with plasma proteins depleted of 12 abundant proteins to improve coverage and digested with trypsin into peptides, which were labeled with amino group-reactive tandem mass tags (TMT) to facilitate quantitative determination of relative expression levels in AP patients versus controls. Here, we report several proteins significantly elevated or decreased in the AP compared to the control subjects. Our findings substantiate the recent development of CRP as a valid biomarker of AP that is superior to more classic biomarkers such as pancreatic lipase. While our data specifically adds validity to the use of CRP as a biomarker of alcoholic AP, they also strongly implicate acute phase proteins in general, as 16 distinct “positive acute phase” proteins (SAA, ORM, AGT, LBP, CRP, FGA, FGB, FGG, SERPINA3, F8, SERPINA1, C9, C1, ITIH3, vWF, and SERPING1), and two “negative acute phase” proteins (Ritchie et al., 1999) (ALB and TF) emerged in our list of proteins elevated and decreased, respectively in alcoholic AP. In addition, several proteins significantly elevated or decreased in AP patients that do not correspond to the acute phase response may represent novel AP biomarkers. Using our list of significantly altered proteins in plasma, we also performed IPA to determine pathobiologic regulatory pathways in AP patients.

## Materials and Methods

### Study Design and Patient Population

These samples arise from a prospective observational study conducted at the Department of Surgery at Kaunas University of Medicine Hospital (Lithuania). Patients admitted during 2006–2007 with a diagnosis of AP, history of significant alcohol intake and onset of the disease within last 72 h were included in this study (Dambrauskas et al., 2010). Plasma from healthy controls was obtained during the same dates by the medical team using similar procedures used in patients. The Regional Ethics Committee approved the study (Protocol Nos. BE-2-47 and P1-113/2005) and all the patients and healthy controls provided written informed consent. The AP diagnosis was established on the basis of acute abdominal pain consistent with AP; and at least threefold elevated levels of serum amylase or typical radiological findings of AP. A contrast-enhanced CT scan was performed on days 4 to 7 after onset of the disease to determine the presence of pancreatic necrosis. Patients with underlying chronic pancreatitis and patients with AP referred to the hospital from other institutions after management for more than 3 days were excluded from this study. Age- and sex-matched healthy subjects (*n* = 18) without previous medical history were enrolled as controls. Volunteers were excluded from participation on the basis of the presence of any illness or any recent surgical procedure.

#### Composite Clinical Scores

For each participant, the APACHE II scores were those calculated on the day of admission. Similarly, the Marshall multiple organ dysfunction syndrome (MODS) score reported was that calculated initially. Severity was assessed according to the clinical course and clinical scores (APACHE II > 7; Imrie-Glasgow > 2; MODS > 2). Clinical data relating to the severity of disease, development of organ dysfunction and/or septic complications were prospectively collected in a standardized fashion. The plasma sample Serum CRP and white blood cells were measured to assess the inflammatory burden of the cohort.

### Sample Acquisition and Handling

Peripheral blood samples from patients were drawn on admission to the hospital and, after centrifugation, plasma samples were stored at −80°C until analysis. The blood samples of control group underwent a similar process. Plasma samples were transferred to Cedars-Sinai Medical Center, Los Angeles, CA, United States where the proteomic analysis was performed. All the procedures were approved by the Cedars-Sinai Medical Center IRB, under protocol Pro00048082.

### Plasma Depletion of Abundant Proteins

Selected AP and control plasma samples were thawed on ice, vortexed to mix and centrifuged for 10 min at 14,000 rpm at 4°C in a microfuge to float lipid and pellet any debris. Ten μl of each cleared plasma was depleted of 12 Abundant proteins using Proteome Purify 12 (R&D Biosystems) immunodepletion resin (Krishnan et al., 2017) according to the manufacturer’s instructions, and the recovered protein solution was acetone-precipitated by combining with 5 volumes of acetone and storing at −20°C for 16 h. This process is designed to remove quantities of α1-Acid Glycoprotein, α1-Antitrypsin, α2-Macroglobulin, Albumin, Apolipoprotein A-I, Apolipoprotein A-II, Fibrinogen, Haptoglobin, IgA, IgG, IgM and Transferrin, but also leaves residual amounts of these proteins, as the removal is not quantitative (not shown). Protein precipitate was recovered by centrifuging at 14,000 rpm at 4°C for 30 min, washed with 50% acetone, centrifuged again and dissolved in 0.1 M Tris-HCl, pH 7.5 with 4% SDS. Protein concentrations were measured using a BCA microassay and all samples adjusted to the lowest common concentration, 0.7 μg/μl.

### Filter-Assisted Sample Preparation Combined With TMT Labeling (iFASP) for Proteomic Analysis

Filter-assisted sample preparation (Wisniewski, 2017) was carried out with the 12 AP samples and 12 controls, as follows: 90 μl (i.e., 60 μg) of each sample was transferred to filter units (Millipore Ultrafree-MC 30,000 Nominal Molecular Weight Limit, low binding regenerated cellulose), the reducing agent TCEP was added to 10 mM, and the tubes placed on a ThermoMixer and heated to 55°C for 1 h with occasional mixing. Then, 8 M urea in 50 mM TEAB (UT) Buffer (300 μl) was added, the samples inverted to mix, and buffer containing excess reducing agent and SDS was depleted from the sample by centrifuging the soluble components through the filter bed at 10,000 rpm on a microfuge at RT. When samples in the filters reached ∼50 μl, they were washed again with 300 μl UT Buffer. To alkylate proteins, 50 mM iodoacetic acid was added. The samples were mixed in the Thermomixer for 1 min, then placed in the dark for 30 min. One wash with UT buffer was followed by two washes with 50 mM TEAB buffer (200 μl each). Proteomic-grade trypsin (Thermo Fisher) was dissolved in the buffer provided (20 μl), then combined with 50 mM TEAB, and 85 μl of this solution was transferred to each filter to give a final trypsin:protein ratio of 1:40. The filters were sealed with Parafilm, then shaken at 600 rpm with temperature set to 37°C for 18 h.

**FIGURE 1:**
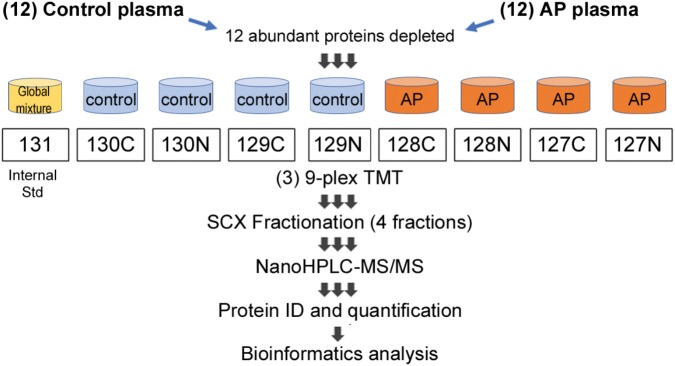
Proteomic workflow for marker identification in alcoholic AP plasma samples. Samples were processed as described in Section “Materials and Methods” and tryptic peptides labeled with amine-reactive tandem mass tags and assembled into nine-plexes as shown. Triplicates of a master mix of all peptides were constructed, labeled with TMT-126 and incorporated into nine-plexes together with four controls and four alcoholic AP plasma samples. As there were 12 controls and 12 alcoholic AP plasma samples, three nine-plexes were needed. Each nine-plex was fractionated into four subfractions and then the 12 samples run sequentially in the mass spectrometry experiment. Data were compiled, proteins identified and quantitated using Proteome Discoverer software. Bioinformatic analysis was performed subsequently using online DAVID software and using IPA software licensed to CSMC and as described in the text.

Following trypsinization, sample volumes were measured, and 0.125 volume of each sample was removed and combined to make a “master mix” or reference standard, which was divided and transferred to three new filters. TMT labeling according to the scheme shown in **Figure 1** was performed according to the manufacturer’s instructions, while peptides were still in the columns, an innovation “iFASP” introduced by the Steen lab (McDowell et al., 2013) to the established FASP method (Wisniewski et al., 2009). Labeled peptides were then spun through the filters, with extraction assisted by twice adding 40 μl 50 mM TEAB, shaking at 800 rpm for 20 min at 37°C and then spinning the filter tubes to collect labeled peptides in clean LoBind tubes. A final extraction was done using 50 μl of 5 mM NaCl in 50 mM TEAB. All peptides spun through the filter were combined and the volume was reduced to ∼170 μl/tube by vacuum centrifugation.

Samples were combined into three nine-plexes, according to the scheme shown in **Figure 1**. The three nine-plexes of TMT-labeled peptides were desalted using OASIS HLB columns by loading in 1 ml 0.1% FA, washing 10 times with 1 ml, then eluted once each with 30, 50, and 70% acetonitrile and combined. ACN was removed by vacuum centrifugation. Peptides were then fractionated using SCX spin columns (Thermo Fisher Scientific), added to the columns in loading solution (LS) containing 0.1% FA/5% ACN. The flow through was kept as one fraction, then after two washes with LS, further peptides were eluted sequentially using a solution (S2) containing 0.1% FA, 0.5 M KCl and 25% ACN, combined with LS at 25:75, 50:50 and 100:0 (i.e., undiluted S2). Fractions were desalted using disposable C18 spin columns and stored at −20°C.

### LC–MS/MS and Systems Analysis of TMT 9-Plexes

The above SCX fractionation yielded four samples from each of the three 9-plex peptide mixtures. These 12 mass spectrometry samples were analyzed by high-resolution mass spectrometry as follows: fractionated peptides were separated by low-pH RPLC and analyzed by LC–MS/MS using an EASY-nLC 1000 connected to an LTQ Orbitrap Elite Mass Spectrometer (Thermo Scientific). Briefly, peptides were loaded onto a 2-cm trap column (PepMap 100 C18, 75 μm inner diameter, 3 μm particles, 100 Å pore size) and separated by a 50-cm EASY-Spray column (PepMap RSLC C_18_, 75 μm inner diameter, 2 μm particles, 100 Å pore size) heated to 55°C. For low-pH RPLC separation, the mobile phase consisted of 0.1% FA in water (phase A) or acetonitrile (phase B). The LC gradient was 4–24% B over 200 min, 24–50% B over 20 min, and 50–100% B over 5 min at a flow rate of 150 nL/min, followed by 100% B over 15 min at a flow rate of 300 nL/min. Mass spectra were acquired in a data-dependent manner, selecting up to 15 most abundant precursor ions for higher-energy collisional dissociation. The mass resolution for precursor and fragment ions were set at 120,000 and 60,000, respectively. The isolation width was set to 1.5 and the normalized collision energy was set to 40. Pierce BSA Protein digest was used as standard for mass spec calibration. All reagents used were mass spectrometry grade. Data were analyzed using Proteome Discoverer (v2.1), with FDR set to 1% and proteins identified on the basis of at least two unique peptides. Proteins identified were quantitated as the average of four triplicate values generated between individual patients and controls, given as the ratio patient/control as a fold change of patient to control. Student’s *t*-test was performed and *P*-value of 0.05 taken as an indicator of significance. Further systems analysis was also carried out by analyzing data using IPA^1^ (IPA, QIAGEN, Inc.). IPA was performed by inputting a list of proteins elevated and reduced, with associated fold-changes.

### Human Pancreatic Acini and Pancreatic Tissues

Human acini preparations and human pancreatic tissues were used in this study. Primary human pancreatic acini were obtained from cadaveric pancreatic tissues from organ donors. Briefly, pancreata were digested at City of Hope (Duarte, CA, United States) to separate islets from acinoductal trees, as described (Qi et al., 2015). The resulting acini were transferred to Cedars-Sinai Medical Center (Los Angeles, CA, United States) and further prepared and characterized as previously described (Lugea et al., 2017). The study was performed in accordance with regulations and protocols approved by the Institutional Review Boards of the Beckman Research Institute of the City of Hope and the Cedars-Sinai Medical Center (IRB Pro00032114). Briefly, normal pancreatic tissue samples were obtained from a deceased organ donor and by surgical resection from a chronic pancreatitis patient following approved institutional review board procedures (Cedars-Sinai Medical Center, institutional review board Pro00034086). Resected pancreas tissues were examined at the Surgical Pathology Department. Normal tissue samples were collected and snap frozen for subsequent analysis or formalin fixed for histological examination to ensure normal tissue morphology. Similar procedures were followed for tissue sections from cadaveric pancreata.

Acini and pancreatic tissue samples were homogenized in RIPA buffer containing 50 mmol/L Tris (pH 7.4), 150 mmol/L NaCl, 1% deoxycholic acid, 1% Triton X-100, 0.1% SDS and a mixture of protease and phosphatase inhibitors (Roche Applied Science, Basel, Switzerland). Protein extracts were resolved by Western Blot Analysis as indicated below.

### Immunoprecipitation and Western Blot Analysis

To analyze transketolase expression in human pancreas tissue and acinar cells, samples of protein (20 μg/lane) were run in Novex WedgeWell 4–20% Tris-glycine SDS-PAGE mini-gels (Invitrogen). Proteins were separated by electrophoresis and subsequently transferred to nitrocellulose membranes using the XCell SureLock mini electrophoresis cell (Invitrogen). Membranes were blocked with 5% non-fat dry milk in TBS-T and incubated first with a rabbit anti-transketolase primary antibody (sc-67120) from Santa Cruz Biotechnology (diluted 1:400 in TBS-T, overnight at 4°C), and then with an HRP-conjugated, anti-rabbit secondary antibody (Cell Signaling Technologies), at 1:5000 for 1 h at RT and developed using Pierce SuperSignal West Pico chemiluminescence substrate (Thermo Fisher) on a Syngene PXi6 chemiluminescence documentation instrument with GeneSys software. Protein loading control was estimated using an antibody against β-actin (#A1978, Sigma-Aldrich). The molecular weight of transketolase (68 kDa) was estimated using Precision Plus dual color protein markers (Bio-Rad). To analyze transketolase content of plasma, control or AP patient plasma samples previously analyzed by quantitative proteomics (chosen at random) were immunoprecitated using the Catch and Release (v.2.0) Reversible Immunoprecipitation System (Millipore), according to the manufacturer’s instructions. Input protein per reaction included 11 μl of plasma (∼600 μg protein) and 20 μl (i.e., 4 μg) of antibody sc-67120 in 500 μl of dilution/wash solution. Incubation was carried out overnight at 4°C, and antibody capture effected using protein A-decorated beads. After washing three times, beads were extracted with gel-loading buffer, and captured transketolase protein was analyzed by SDS-PAGE and Western blot analysis as described above.

## Results

### Clinical Data

Blood samples were obtained from patients admitted to the hospital with a diagnosis of AP and an onset of the disease within the last 72 h. The diagnosis of AP was made based on typical abdominal pain, at least threefold elevated levels of serum amylase, and typical radiological findings. Two of three criteria were needed to make a diagnosis of AP, and peripheral blood samples were drawn on admission. The severity of AP was evaluated at 48 h after presentation.

For this study, we tested 12 human AP plasma samples. The primary selection criteria were elevated LDH (>400 IU/L) (see **Table 1**) and a history of alcohol abuse as an etiologic factor. Patients with age < 20 or > 70 years-old were excluded. Age and sex matched healthy subjects (*n* = 12) without previous medical history were enrolled into the control group. Selected AP patients were all males with alcohol as potential etiologic factor (according to the patients self-reporting of alcohol abuse). As indicated in **Table 2**, the average age of AP patients was 40.3 year (median = 39.0); LDH levels, 1172.6 IU/L; APACHE II score, 5.4; IMRIE score, 3.5; and MODS score, 3.3. Average duration of treatment was 15.2 days (median = 16.5). Complications included: IAP (27 mmHg) in one AP patient that died during hospitalization; another patient had MODS, heart failure and renal insufficiency; and two patients had diabetes. No surgical interventions were needed; one patient required ERCP, and two patients required ultrasound guided drainage.

**Table 1:** Acute pancreatitis severity scores^∗^.

	Number of patients	Mean	Standard deviation	Median	Minimum	Maximum
Age (years)	12	40.3	10.4	39	25	66
LDH (U/L)	12	1172.6	735.6	1077.5	420.0	3028.0
APACHE II	12	5.4	4.1	5.5	0	11.0
IMRIE	12	3.5	1.8	3.5	1.0	6.0
MODS	12	3.3	2.7	3.0	0	10.0

*^∗^For each participant, the scores were calculated at 48 h after presentation.*

**Table 2:** Plasma markers of ethanolic AP from quantitative proteomics.

Accession	Name/gene name	MW (kDa)	Unique peptides	Coverage (%)	Avg fold	*t*-Test (*P*-value)
**Proteins increased in alcoholic AP**						
P02741	C-reactive protein/CRP	25	8	28	7.48	0.00
P29401	Transketolase/TKT	68	3	6	6.29	0.00
P02750	Leucine-rich alpha-2-glycoprotein/LRG1	38	10	30	4.03	0.01
A0A096LPE2	Protein SAA2-SAA4/SAA2-SAA4	23	6	42	3.96	0.01
P05109	Protein S100-A8/S100A8	11	5	45	3.78	0.01
P06702	Protein S100-A9/S100A9	13	3	25	3.33	0.05
P01011	Alpha-1-antichymotrypsin/SERPINA3	48	21	43	3.10	0.00
Q08830	Fibrinogen-like protein 1/FGL1	36	3	13	3.08	0.02
P02679	Fibrinogen gamma chain/FGG	51	28	58	3.06	0.02
P02675	Fibrinogen beta chain/FGB	56	38	71	2.96	0.02
P02763	Alpha-1-acid glycoprotein 1/ORM1	23	5	35	2.91	0.01
P04275	von Willebrand factor/VWF	309	12	5	2.82	0.00
P01009	Alpha-1-antitrypsin/SERPINAI	47	28	66	2.71	0.01
P02671	Fibrinogen alpha chain/FGA	95	42	42	2.66	0.04
P0DJI8	Serum amyloid A-1 protein/SAA1	14	3	47	2.41	0.03
P13796	Plastin-2/LCP1	70	8	15	2.39	0.03
Q9UK55	Protein Z-dependent protease inhibitor/SERPINA10	51	5	10	1.98	0.00
P18428	Lipopolysaccharide-binding protein/LBP	53	6	14	1.86	0.01
P19652	Alpha-1-acid glycoprotein 2/ORM2	24	4	26	1.81	0.05
P07195	L-Lactate dehydrogenase B chain/LDHB	37	2	11	1.79	0.01
P00740	Coagulation factor IX/F9	52	8	17	1.78	0.00
P04040	Catalase/CAT	60	8	16	1.76	0.00
P08571	Monocyte differentiation antigen CD14/CD14	40	4	9	1.70	0.00
Q06033	Inter-alpha-trypsin inhibitor heavy chain H3/ITIH3	100	13	16	1.69	0.03
P02748	Complement component C9/C9	63	23	37	1.57	0.05
P05155	Plasma protease C1 inhibitor/SERPING1	55	14	26	1.54	0.01
P07998	Ribonuclease pancreatic/RNASEI	18	2	19	1.53	0.03
P00736	Complement C1r subcomponent/C1R	80	17	27	1.53	0.02
P01019	Angiotensinogen/AGT	53	8	18	1.50	0.02
**Proteins decreased in alcoholic AP**						
P80108	Phosphatidylinositol-glycan-specific phospholipase D/GPLD1	92	11	13	0.63	0.01
P02775	Platelet basic protein/PPBP	14	3	26	0.61	0.02
Q9UGM5	Fetuin-B/FETUB	42	6	16	0.60	0.02
P02787	Serotransferri n/T F	77	48	58	0.54	0.02
P05154	Plasma serine protease inhibitor/SERPINA5	46	5	14	0.47	0.00
P02768	Serum albumin/ALB	69	76	87	0.46	0.02

### Proteomic Analysis Reveals Altered Levels of Several Proteins in Plasma From AP Patients Compared to Controls

**FIGURE 2:**
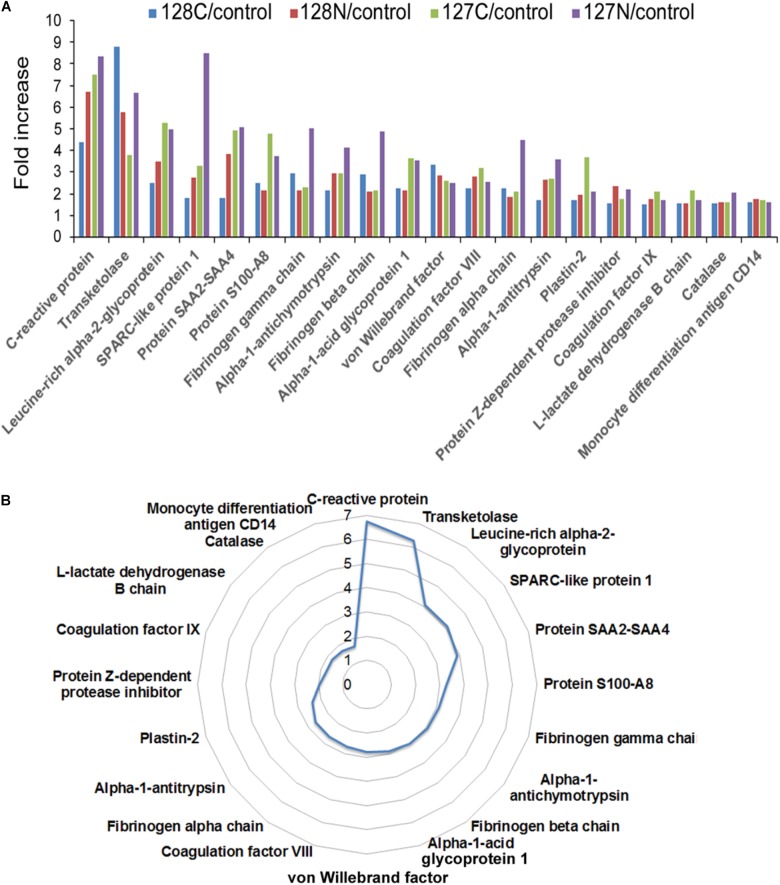
**(A)** Bar chart depicting proteins with quadruplicate elevated levels in alcoholic AP patient plasma. All proteins significantly elevated above 1.5-fold in AP plasma in all four values returned by the analysis are presented with all of the individual values displayed. **(B)** Circular chart showing the same proteins with the averaged values shown. The circular chart illustrates a gradual increase in the proteins closer to 1.5-fold, and big jumps to higher levels with a few outliers at the top end of the fold-changes. Some proteins were also decreased in the analysis. Both increased and decreased proteins can be found in **Table 2**.

The experimental design schematically illustrating the composition of the multiplex proteomic sample labeling with TMT and assembly into three nine-plexes is shown in **Figure 1**. Tandem mass spectrometry and Proteome Discoverer analysis identified 4217 peptides and 285 proteins. Many proteins were changed in the AP patients relative to controls, as shown by mean values of fold change returned by the analysis. To generate a list of proteins increased or decreased, up to four determined values were possible for each protein (i.e., one from each of four fractions). The top proteins (by average fold change) as shown in **Figure 2A**, often elevated in all four samples, were analyzed by *t*-test. Fold-changes of 1.5-fold or more or 0.67-fold or less were included, and we report here only those for which *P* = 0.05 or less as a measure of statistical significance (**Table 2**). A separate graphic in **Figure 2B** also depicts these proteins in a circular fashion to highlight those with the highest elevated levels.

### Gene Ontology and Ingenuity Pathway Analysis (IPA)

**FIGURE 3:**
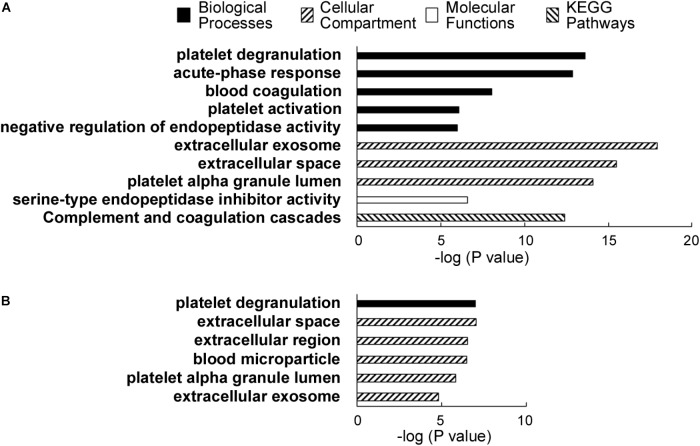
**(A)** DAVID analysis results showing the major categories of gene ontology (GO) associated with the proteins elevated in alcoholic AP patient plasma. A legend shows the categories, i.e., biological processes, cellular compartments, and molecular functions and KEGG pathways. **(B)** DAVID results showing the associated GO categories associated with proteins decreased in the alcoholic AP patients’ plasma.

Gene Ontology analysis was performed using the 31 significantly elevated or the six decreased proteins as input (**Table 2**). Thus, according to analysis using the Database for Annotation, Visualization and Integrated Discovery (DAVID) software (Huang et al., 2009a,b) version 6.8, the major gene ontology categories (Biological Processes, Cellular compartments and Molecular Functions) and Kyoto Encyclopedia of Genes and Genomes (KEGG) pathways detected are described in **Figure 3**.

Notable biological processes associated with proteins elevated in AP samples include platelet degranulation, acute phase response, blood coagulation, platelet activation, and negative regulation of endopeptidase activity. Notable cellular compartments associated with the elevated proteins include exosomes, extracellular space, and platelet alpha granule lumen. The most closely associated molecular function with the proteins elevated in AP was serine-type endopeptidase inhibitor activity, due to the presence of multiple SERPINs, ITIH3 and AGT, a non-inhibitory member of the SERPIN family (Heit et al., 2013). ITIH3 was recently reported as a PDAC serum biomarker (Liu et al., 2017).

According to DAVID analysis, nearly all (29 of 31) the increased proteins have been associated with or detected in the cellular compartment of exosomes. Exosomes are secreted particles initiated by invagination of late endosomal boundary membrane and packaged by the ESCRT pathway into multivesicular bodies. Overall, whereas the proteins increased in ethanolic AP plasma corresponded to a wide range of blood functions, compartments and processes, including ordinary complement, fibrinolysis and blood coagulation components normally secreted to the extracellular space, there is also evidence of pathologic issues such as platelet degranulation and the acute phase response, in addition to both innate and adaptive immunity. We note that some of the proteins differentially elevated or decreased in our TMT-based quantitative analysis are among the 12 depleted proteins. These include α1-antitrypsin/SERPINA1, Albumin and Transferrin. However, as these proteins were depleted consistently from all samples, it is unlikely that these differences arose from the depletion process used in our sample preparation workflow. A recent article that used the same abundant plasma protein removal procedure had a similar conclusion (Krishnan et al., 2017).

**FIGURE 4:**
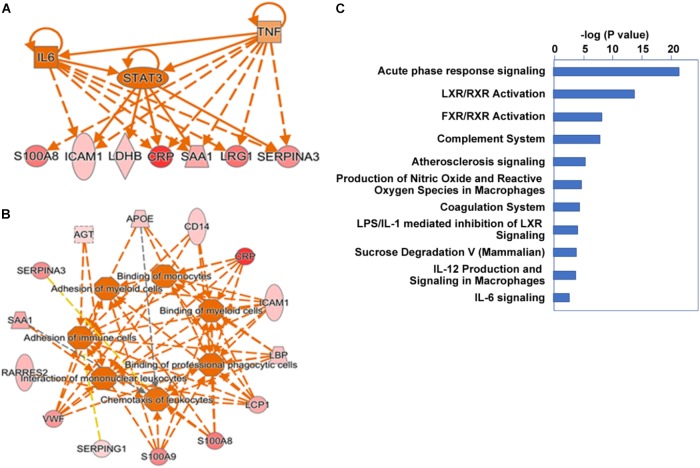
Ingenuity pathway analysis cascading upstream cytokine and transcription factor regulatory pathways **(A)** and downstream pathobiologic effectors **(B)** predicted from our proteomic dataset. Dark orange implies a predicted mediator or process. Red intensity shows expression level. **(C)** Major canonical pathways associated with the gene set.

We further extended our systems analysis of the ensemble of proteins elevated and reduced in alcoholic AP by IPA analysis. Proteins (Uniprot IDs) were input into the analysis together with up or down fold-changes. Results of this analysis are shown in **Figure 4**. IPA analysis predicted that many of the acute phase proteins are synthesized via an upstream signal transduction network involving cytokines such as TNFα, IL-1, IL-6 and OSM, and key transcription factors such as STAT3 (see **Figure 4A**). Other transcription factors such as NF-κB, AP-1 and NF-IL6, and nuclear factors such as corticosteroid-GCR also play important roles in the acute phase signaling pathway network (data not shown). These signal transduction pathways initiated in hepatocytes and other cells induce many downstream events including chemotaxis, binding and invasion of inflammatory cells, as shown schematically in **Figure 4B**. Elevation of genes such as angiotensinogen and apolipoproteins E and C2 were also predictive of upstream activation of LXR/RXR signaling (not shown). A summary of the associated canonical pathways and *P*-values as a measure of their relative statistical significance is shown in **Figure 4C**. In AP, many other cytokines, chemokines, and signaling molecules may also be involved in triggering various responses which systematically account for SIRS and organ failure (Dambrauskas et al., 2010). We anticipate that insights into the upstream pathways triggering AP such as those produced by IPA analysis will give rise to targeted treatments for AP.

### Validation of Selected Proteins Found Elevated in Plasma From AP Patients Compared to Controls

Among the distinct proteins found elevated in the AP samples (shown in **Figure 2** and **Table 2**), CRP had the highest fold-change, measured as 7.5-fold higher in AP than control samples. As mentioned above, CRP has been studied and found to be an excellent indicator of AP severity, although it can be elevated in other inflammatory conditions (Komolafe et al., 2017). Its use in the clinic is increasing due to availability of an inexpensive test (Meher et al., 2015), although its diagnostic accuracy may depend on the timing and the clinical utility of CRP or other biomarkers for prediction remains uncertain (Komolafe et al., 2017). Whereas most of the proteins we found elevated in AP are secreted proteins, the second highest protein, transketolase is an intracellular enzyme. Leucine-rich α2 glycoprotein (LRG1) has been identified as a marker of granulocyte differentiation, is involved in neovascularization by altering TGFα signaling at endothelial cells (Wang et al., 2013), and has recently been found elevated in plasma associated with distinct cancers including pancreatic (Capello et al., 2017). SPARC-like protein 1, also known as HEVIN, is described as a matricellular protein, which may have positive and/or negative roles in distinct cancers (Gagliardi et al., 2017). S100A8, or calprotectin is a small calcium-binding protein often associated with immune cells (Gebhardt et al., 2006). Plastin-2 or Plastin-L/LCP1 is an actin-binding protein associated with immune cells and may be elevated in epithelial cells undergoing tumorigenesis (Shinomiya, 2012). Among the other elevated proteins, one that has been widely hypothesized to play distinct roles in pancreatic diseases is angiotensinogen (Skipworth et al., 2011).

**FIGURE 5:**
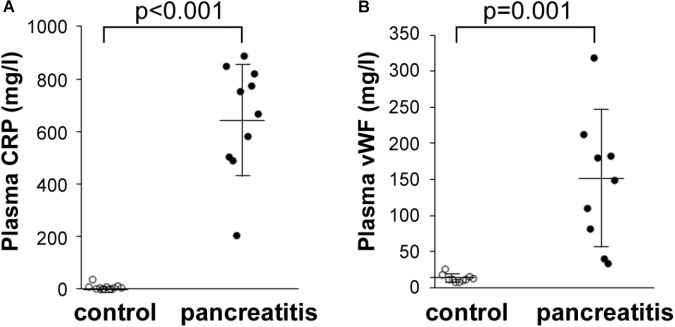
Luminex determination of **(A)** CRP and **(B)** vWF in control and alcoholic AP patient plasma samples. Samples were thawed, diluted 40,000-fold as instructed by the manufacturer, and processed further according to instructions. Readings were taken from duplicate measurements, and controls versus alcoholic AP patient plasma results graphed with the mean and standard deviation shown by the horizontal lines with error bars; *n* = 9–10. Statistical significance was evaluated by Student’s *t-*test. *P*-value for CRP measurement in AP patients was < 0.001. *P*-value for vWF was = 0.001.

We also performed additional laboratory tests to reevaluate the levels of CRP, as well as another acute phase protein found elevated in the AP patients’ sera by quantitative mass spectrometry, vWF. To achieve this, we performed Luminex assays in a number of the same plasma samples analyzed by quantitative LC–MS/MS. Shown in **Figure 5A**, CRP levels in all the AP plasma samples were dramatically elevated compared with those in control plasma samples. Similarly, vWF levels were also elevated, but to a lesser extent than CRP (**Figure 5B**). These data indicate that a highly sensitive and exacting test such as Luminex provides a similar series of elevated levels as that reported by TMT labeling/LC–MS/MS, i.e., CRP > vWF. These data thereby independently validate our finding of uniform elevation of these two acute phase proteins in the AP patient plasma samples analyzed.

**FIGURE 6:**
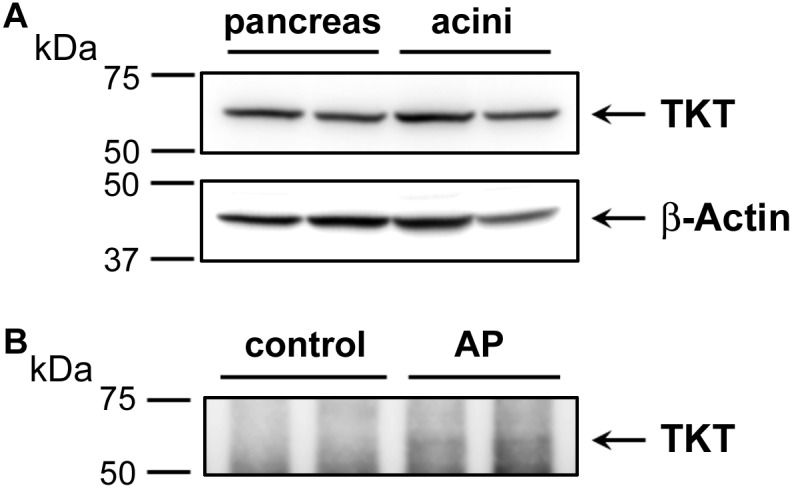
Western blot analysis depicting the expression of transketolase (TKT) in human pancreas and acinar cell samples **(A)** and as immunoprecipitated from control versus alcoholic AP patients’ plasma **(B)**. In **(A)** approximately 20 μg of protein from each of two samples of human pancreas homogenate (left) and two samples of human acinar cell samples (right) were analyzed for TKT expression (upper panel). The same representative blot was reprobed for β-actin as loading control (lower panel). In **(B)** plasma samples (∼600 μg) were subjected to IP using 4 μg anti-TKT antibody (*n* = 2). Arrows indicate the position of TKT.

Elevated plasma transketolase (TKT), to our knowledge has not been previously reported as an AP biomarker. This enzyme is normally not secreted, although it is expressed in pancreas and may be released from damaged pancreatic tissue. Here, we also performed Western blot analysis to detect TKT expression. We first showed that a commercial antibody was successful to detect TKT in samples of human pancreas tissue and isolated human acinar cells (**Figure 6**). These data show the efficacy of the antibody and the substantial expression in pancreas. We next used the antibody to isolate elevated plasma TKT by immunoprecipitation from a subset of proteomically analyzed AP plasma samples. Thus, full length TKT was immunoprecipitated from two control and two AP samples. Results of Western blot analysis carried out with these immunoprecipitated samples, shown in **Figure 6** demonstrate that, whereas there is no detectable TKT in control plasma immunoprecipitates, bands selectively appear in AP samples. These data provide further evidence which substantiates our finding of increased TKT in the AP patients’ plasma by mass spectrometry.

## Discussion

The presence of elevated levels of proteins identified in this study, including a significant number of acute phase response proteins may be indicative of distinct disease processes and risk for severe inflammatory disease complications and cancer. Further study is needed to determine the specificity and selectivity of these proteins as biomarkers in alcoholic AP. Acute phase proteins appear to be well-suited to confirm the presence of systemic inflammatory process in AP and individual proteins such as CRP will likely continue to be used clinically. However, these proteins may also be elevated in other disease processes, and therefore they lack specificity. More novel is our finding of elevated TKT, the cellular source of which is unknown. Measurement of erythrocyte TKT activity has been used for many years as a measure of thiamine deficiency (Thomson et al., 2010). Also, erythrocyte abnormalities and hemolysis are known to occur in alcoholics (Ballard, 1997). Further, another study found that TKT protein can be diminished in hemolysates from alcoholic subjects (Takeuchi et al., 1988). Taken together, these considerations suggest erythrocytes as a possible source of elevated plasma TKT in alcoholic AP patients.

However, our previous study undertaken with proteomic analysis of normal human pancreas tissue also indicate that TKT is highly expressed in pancreas (Lugea et al., 2017). Thus, excess TKT in plasma could also be evidence of pancreas damage. Further studies will be needed to evaluate whether TKT elevation is unique to alcoholic AP, is found more generally elevated in AP, or is useful as a measure of predicted severity in these or other patients. An important metabolic role was recently ascribed to TKT to promote pancreatic cancer development through conversion of fructose to nucleotides for anabolic metabolism (Liu et al., 2010). Elevation of LRG1, SPARCL1, S100A8, Plastin-2 may be of special significance in a small but significant percentage of patients which have increased risk for prolonged development of a disease spectrum that connects AP, RAP, and chronic pancreatitis with emergence of pancreatic cancer. Pancreatic cancer exhibits profound alterations in neovascularization, which likely precede and may even be pivotal in tumor initiation in the context of CP. Thus, findings reported here may have added significance in increasing our understanding of the poorly-understood links between pancreatitis and pancreatic cancer.

The use of IPA as an adjunct to genomic and proteomic investigations is rising steadily and has been included in over 2000 published papers in PubMed, 42 of which touch upon some aspect of pancreas or pancreatic research. IPA relies on a sophisticated, comprehensive manually curated information base, a strength of which is its ability to factor in whether the level of each gene increases or decreases. This allows data to be integrated, interpreted, and displayed in the context of previously documented biological pathways. In one study, different oncogenes were introduced into a normal ductal cell background, and IPA was used to understand which tumorigenic pathways were initiated in the cells (Chang et al., 2013). More recently, IPA was used to interpret effects of epigenetic reprogramming in Mist1−/− mice (Mehmood et al., 2014). Here, IPA showed that many of the proteins elevated during alcoholic pancreatitis are those of the acute phase response. Beyond this, some of the key transcription factors and chemokines/cytokines involved were pinpointed, giving indications as to which pathways are activated and might be targeted in future studies, or even therapeutically. It has become clear that incorporating IPA and other bioinformatic approaches into studies that generate a set of genes or proteins has great potential to enhance our understanding of how normal biology is disrupted in disease processes.

## Conclusion

Given the wide range of outcomes in AP, and the failure of clinical scoring systems to adequately prepare caregivers for different outcomes in the AP disease course, much research has been dedicated to identification of stand-alone protein biomarkers which would be of value in assessing the severity of AP patients. Nevertheless, whereas several potential markers of severity including CRP have been tested to a limited extent, none have been established as standards of care for this purpose. Here, we used a shotgun proteomics approach to search for proteins elevated or decreased in a small group of alcoholic AP patients versus controls. This approach resulted in the identification of CRP as the most elevated protein in these plasma samples, which validates our approach since this is the protein most often regarded as a valuable early AP biomarker. Other increased proteins including transketolase are novel and will require further study. Some proteins were also selectively decreased in alcoholic AP samples in this study. These may also be significant in pancreas diseases as well as in various other inflammatory diseases.

## Author Contributions

RW performed the proteomic and other experiments and bioinformatic analyses, analyzed and interpreted data with all other authors and drafted the manuscript and figures. AL analyzed and interpreted data and revised the manuscript and figures. AG procured samples and clinical information, initiated the study, and revised the manuscript. SP interpreted data and revised the manuscript.

## Conflict of Interest Statement

The authors declare that the research was conducted in the absence of any commercial or financial relationships that could be construed as a potential conflict of interest.

## Footnotes

1 https://www.qiagenbioinformatics.com/products/ingenuity-pathway-analysis/

## Abbreviations

ACN: acetonitrile
AGT: angiotensinogen
ALB: serum albumin precursor
AP: acute pancreatitis
APACHE: Acute Physiology and Chronic Health Examination
BCA: bicinchoninic acid
C1: complement C1
C9: complement C9
CRP: C-reactive protein
ERCP: endoscopic retrograde cholangiopancreatography
ESCRT: endosomal sorting complex required for transport
F8: coagulation factor VIII
FA: formic acid
FGA: fibrinogen alpha chain
FGB: fibrinogen beta chain precursor
FGG: fibrinogen gamma chain precursor
GCR: glucocorticoid receptor
IAP: intra-abdominal pressure
IL1: interleukin-1
IL6: interleukin-6
IMRIE: modified Glasgow Imrie severity criteria for acute pancreatitis
IPA: ingenuity pathway analysis
ITIH3: inter-alpha-trypsin inhibitor heavy chain 3
LBP: lipopolysaccharide binding protein
LC–MS/MS: liquid chromatography–tandem mass spectrometry
LDH: lactate dehydrogenase
LPS binding protein: lipopolysaccharide binding protein
LRG1: leucine-rich alpha-2-glycoprotein 1
MODS: multiple organ dysfunction syndrome
NFIL6: nuclear factor for IL-6 expression
ORM: alpha-1-acid glycoprotein 1
OSM: oncostatin M
RAP: recurrent acute pancreatitis
RT: room temperature
S100A8: S100 calcium-binding protein A8
SAA: serum amyloid A
SCX: strong cation exchange
SDS-PAGE: sodium dodecylsulfate-polyacrylamide gel electrophoresis
SERPINA1: alpha-1-proteinase inhibitor
SERPINA3: alpha-1-antichymotrypsin
SERPING1: plasma protease C1 inhibitor precursor
SIRS: systemic inflammatory response syndrome
SPARCL1: secreted protein, acidic and rich in cysteine-like 1
TBS-T: Tris-buffered saline supplemented with 0.075% Tween-20
TEAB: triethylammonium bicarbonate
TF: transferrin
TKT: transketolase
TMT: tandem mass tag
TNF: tumor necrosis factor
vWF: von Willebrand factor.
